# Supplementary material for: Modeling glioblastoma heterogeneity as a dynamic network of cell states
Source: Mol Syst Biol. 2021 Sep 16;17(9):e10105. doi: 10.15252/msb.202010105 (PMC8444284; doi:10.15252/msb.202010105)
Supplement: Supplementary file 6 — Source Data for Figure 5 [file MSB-17-e10105-s004.zip › Figure5A_sourcedata/GSEA_3071/nosigdb_stateII.GseaPreranked.1622795151171/FELDMAN_2020_S-G2.html]

Details for gene set FELDMAN\_2020\_S-G2[GSEA]

|  || Dataset | state23071 |
| Phenotype | NoPhenotypeAvailable |
| Upregulated in class | na\_pos |
| GeneSet | FELDMAN\_2020\_S-G2 |
| Enrichment Score (ES) | 0.59078944 |
| Normalized Enrichment Score (NES) | 4.273918 |
| Nominal p-value | 0.0 |
| FDR q-value | 0.0 |
| FWER p-Value | 0.0 |
Table: GSEA Results Summary

  

Fig 1: Enrichment plot: FELDMAN\_2020\_S-G2      
 Profile of the Running ES Score & Positions of GeneSet Members on the Rank Ordered List

  

| PROBE | GENE SYMBOL | GENE\_TITLE | RANK IN GENE LIST | RANK METRIC SCORE | RUNNING ES | CORE ENRICHMENT || 1 | HIST1H1D |  |  | 0 | 2.323 | 0.0095 | Yes |
| 2 | CLSPN |  |  | 1 | 1.984 | 0.0176 | Yes |
| 3 | HIST1H4C |  |  | 2 | 1.902 | 0.0254 | Yes |
| 4 | RRM2 |  |  | 3 | 1.857 | 0.0330 | Yes |
| 5 | ANLN |  |  | 4 | 1.760 | 0.0402 | Yes |
| 6 | IL11 |  |  | 5 | 1.740 | 0.0473 | Yes |
| 7 | HIST1H1B |  |  | 7 | 1.669 | 0.0539 | Yes |
| 8 | TYMS |  |  | 8 | 1.665 | 0.0607 | Yes |
| 9 | CDK1 |  |  | 14 | 1.540 | 0.0657 | Yes |
| 10 | DIAPH3 |  |  | 15 | 1.531 | 0.0720 | Yes |
| 11 | ZWINT |  |  | 16 | 1.510 | 0.0782 | Yes |
| 12 | HJURP |  |  | 17 | 1.490 | 0.0843 | Yes |
| 13 | ATAD5 |  |  | 18 | 1.469 | 0.0903 | Yes |
| 14 | FEN1 |  |  | 19 | 1.464 | 0.0963 | Yes |
| 15 | HIST2H2AC |  |  | 20 | 1.462 | 0.1022 | Yes |
| 16 | BRCA2 |  |  | 22 | 1.445 | 0.1079 | Yes |
| 17 | UBE2C |  |  | 23 | 1.438 | 0.1138 | Yes |
| 18 | CENPU |  |  | 24 | 1.416 | 0.1196 | Yes |
| 19 | RAD51AP1 |  |  | 25 | 1.384 | 0.1252 | Yes |
| 20 | CENPK |  |  | 26 | 1.382 | 0.1309 | Yes |
| 21 | CDCA4 |  |  | 27 | 1.376 | 0.1365 | Yes |
| 22 | MYBL2 |  |  | 28 | 1.362 | 0.1421 | Yes |
| 23 | FBXO5 |  |  | 30 | 1.359 | 0.1474 | Yes |
| 24 | USP1 |  |  | 31 | 1.357 | 0.1530 | Yes |
| 25 | TCF19 |  |  | 32 | 1.331 | 0.1584 | Yes |
| 26 | PSMC3IP |  |  | 36 | 1.322 | 0.1630 | Yes |
| 27 | RRM1 |  |  | 37 | 1.310 | 0.1684 | Yes |
| 28 | ORC6 |  |  | 39 | 1.308 | 0.1735 | Yes |
| 29 | GINS2 |  |  | 40 | 1.306 | 0.1788 | Yes |
| 30 | TK1 |  |  | 42 | 1.281 | 0.1838 | Yes |
| 31 | AURKB |  |  | 44 | 1.276 | 0.1888 | Yes |
| 32 | MCM7 |  |  | 45 | 1.273 | 0.1940 | Yes |
| 33 | HELLS |  |  | 46 | 1.259 | 0.1991 | Yes |
| 34 | HIST1H2AH |  |  | 50 | 1.249 | 0.2035 | Yes |
| 35 | CCNA2 |  |  | 53 | 1.230 | 0.2080 | Yes |
| 36 | DNAJC9 |  |  | 56 | 1.217 | 0.2124 | Yes |
| 37 | SKA3 |  |  | 57 | 1.215 | 0.2174 | Yes |
| 38 | PCNA |  |  | 58 | 1.214 | 0.2224 | Yes |
| 39 | CDC45 |  |  | 59 | 1.212 | 0.2273 | Yes |
| 40 | HIST1H1C |  |  | 60 | 1.206 | 0.2323 | Yes |
| 41 | MAD2L1 |  |  | 61 | 1.199 | 0.2372 | Yes |
| 42 | UBE2T |  |  | 62 | 1.192 | 0.2421 | Yes |
| 43 | ATAD2 |  |  | 63 | 1.189 | 0.2469 | Yes |
| 44 | SPC25 |  |  | 68 | 1.156 | 0.2506 | Yes |
| 45 | MELK |  |  | 69 | 1.145 | 0.2553 | Yes |
| 46 | TOP2A |  |  | 72 | 1.132 | 0.2594 | Yes |
| 47 | DSN1 |  |  | 75 | 1.122 | 0.2635 | Yes |
| 48 | FOSL1 |  |  | 77 | 1.119 | 0.2678 | Yes |
| 49 | GMNN |  |  | 83 | 1.108 | 0.2710 | Yes |
| 50 | RBBP8 |  |  | 86 | 1.100 | 0.2750 | Yes |
| 51 | CEP55 |  |  | 87 | 1.096 | 0.2795 | Yes |
| 52 | KIFC1 |  |  | 88 | 1.094 | 0.2840 | Yes |
| 53 | HIST1H1E |  |  | 90 | 1.088 | 0.2881 | Yes |
| 54 | VRK1 |  |  | 93 | 1.074 | 0.2920 | Yes |
| 55 | NDC80 |  |  | 94 | 1.074 | 0.2964 | Yes |
| 56 | LRR1 |  |  | 98 | 1.060 | 0.3000 | Yes |
| 57 | TMPO |  |  | 99 | 1.056 | 0.3043 | Yes |
| 58 | NCAPG |  |  | 100 | 1.056 | 0.3086 | Yes |
| 59 | DHFR |  |  | 101 | 1.051 | 0.3129 | Yes |
| 60 | MND1 |  |  | 104 | 1.036 | 0.3166 | Yes |
| 61 | MCM3 |  |  | 105 | 1.035 | 0.3209 | Yes |
| 62 | MKI67 |  |  | 107 | 1.031 | 0.3248 | Yes |
| 63 | TPX2 |  |  | 111 | 1.025 | 0.3282 | Yes |
| 64 | RFC2 |  |  | 112 | 1.022 | 0.3324 | Yes |
| 65 | TRIP13 |  |  | 114 | 1.014 | 0.3363 | Yes |
| 66 | DUT |  |  | 118 | 1.009 | 0.3396 | Yes |
| 67 | ASF1B |  |  | 120 | 1.007 | 0.3435 | Yes |
| 68 | SMC2 |  |  | 123 | 1.005 | 0.3471 | Yes |
| 69 | C12orf75 |  |  | 124 | 1.004 | 0.3512 | Yes |
| 70 | NUF2 |  |  | 125 | 1.004 | 0.3553 | Yes |
| 71 | DEPDC1 |  |  | 126 | 0.999 | 0.3594 | Yes |
| 72 | CKS1B |  |  | 128 | 0.999 | 0.3632 | Yes |
| 73 | TACC3 |  |  | 130 | 0.992 | 0.3670 | Yes |
| 74 | KIF20B |  |  | 133 | 0.989 | 0.3705 | Yes |
| 75 | ESCO2 |  |  | 144 | 0.971 | 0.3719 | Yes |
| 76 | PLP2 |  |  | 147 | 0.962 | 0.3753 | Yes |
| 77 | CKAP2L |  |  | 148 | 0.959 | 0.3792 | Yes |
| 78 | RANBP1 |  |  | 149 | 0.958 | 0.3832 | Yes |
| 79 | GGCT |  |  | 160 | 0.937 | 0.3844 | Yes |
| 80 | KIF23 |  |  | 161 | 0.936 | 0.3882 | Yes |
| 81 | DONSON |  |  | 163 | 0.933 | 0.3918 | Yes |
| 82 | SHCBP1 |  |  | 164 | 0.933 | 0.3956 | Yes |
| 83 | RTKN2 |  |  | 166 | 0.931 | 0.3991 | Yes |
| 84 | GTSE1 |  |  | 167 | 0.929 | 0.4029 | Yes |
| 85 | CEP78 |  |  | 170 | 0.927 | 0.4062 | Yes |
| 86 | CDCA2 |  |  | 171 | 0.922 | 0.4100 | Yes |
| 87 | CENPM |  |  | 178 | 0.901 | 0.4121 | Yes |
| 88 | KPNB1 |  |  | 186 | 0.891 | 0.4139 | Yes |
| 89 | SAC3D1 |  |  | 188 | 0.887 | 0.4173 | Yes |
| 90 | SPDL1 |  |  | 199 | 0.874 | 0.4182 | Yes |
| 91 | TFDP1 |  |  | 209 | 0.866 | 0.4194 | Yes |
| 92 | H2AFZ |  |  | 213 | 0.864 | 0.4221 | Yes |
| 93 | PBK |  |  | 214 | 0.864 | 0.4257 | Yes |
| 94 | CDCA3 |  |  | 219 | 0.858 | 0.4281 | Yes |
| 95 | CENPH |  |  | 221 | 0.855 | 0.4314 | Yes |
| 96 | RFC5 |  |  | 222 | 0.851 | 0.4349 | Yes |
| 97 | ASPM |  |  | 231 | 0.842 | 0.4362 | Yes |
| 98 | HMGA1 |  |  | 238 | 0.834 | 0.4380 | Yes |
| 99 | MIS18A |  |  | 239 | 0.833 | 0.4415 | Yes |
| 100 | DBF4 |  |  | 240 | 0.832 | 0.4449 | Yes |
| 101 | PRC1 |  |  | 242 | 0.830 | 0.4480 | Yes |
| 102 | TUBB6 |  |  | 244 | 0.828 | 0.4511 | Yes |
| 103 | C19orf48 |  |  | 248 | 0.826 | 0.4537 | Yes |
| 104 | LMNB1 |  |  | 251 | 0.824 | 0.4566 | Yes |
| 105 | DNMT1 |  |  | 265 | 0.792 | 0.4564 | Yes |
| 106 | TUBB4B |  |  | 271 | 0.784 | 0.4583 | Yes |
| 107 | YWHAH |  |  | 273 | 0.781 | 0.4612 | Yes |
| 108 | HMGB2 |  |  | 276 | 0.780 | 0.4639 | Yes |
| 109 | SMC4 |  |  | 279 | 0.777 | 0.4665 | Yes |
| 110 | EIF2S1 |  |  | 282 | 0.776 | 0.4692 | Yes |
| 111 | HSPB11 |  |  | 283 | 0.775 | 0.4724 | Yes |
| 112 | CENPE |  |  | 284 | 0.775 | 0.4755 | Yes |
| 113 | TEX30 |  |  | 287 | 0.774 | 0.4782 | Yes |
| 114 | DEK |  |  | 302 | 0.754 | 0.4776 | Yes |
| 115 | CENPA |  |  | 303 | 0.751 | 0.4807 | Yes |
| 116 | KIF4A |  |  | 309 | 0.746 | 0.4824 | Yes |
| 117 | COPS3 |  |  | 310 | 0.746 | 0.4855 | Yes |
| 118 | CKS2 |  |  | 312 | 0.745 | 0.4882 | Yes |
| 119 | CENPN |  |  | 320 | 0.737 | 0.4894 | Yes |
| 120 | ANP32B |  |  | 325 | 0.734 | 0.4914 | Yes |
| 121 | CENPW |  |  | 330 | 0.727 | 0.4933 | Yes |
| 122 | PHLDA2 |  |  | 332 | 0.726 | 0.4960 | Yes |
| 123 | WDR34 |  |  | 333 | 0.724 | 0.4990 | Yes |
| 124 | ARHGAP11A |  |  | 346 | 0.712 | 0.4987 | Yes |
| 125 | AKAP12 |  |  | 349 | 0.711 | 0.5011 | Yes |
| 126 | HPRT1 |  |  | 351 | 0.709 | 0.5038 | Yes |
| 127 | KLF6 |  |  | 357 | 0.708 | 0.5054 | Yes |
| 128 | TOMM40 |  |  | 358 | 0.706 | 0.5082 | Yes |
| 129 | DTYMK |  |  | 364 | 0.701 | 0.5098 | Yes |
| 130 | EIF5 |  |  | 366 | 0.701 | 0.5124 | Yes |
| 131 | PSMG1 |  |  | 372 | 0.696 | 0.5139 | Yes |
| 132 | TMEM106C |  |  | 377 | 0.694 | 0.5157 | Yes |
| 133 | SIVA1 |  |  | 380 | 0.690 | 0.5180 | Yes |
| 134 | RBM17 |  |  | 383 | 0.687 | 0.5203 | Yes |
| 135 | RFC4 |  |  | 385 | 0.686 | 0.5229 | Yes |
| 136 | SUPT16H |  |  | 394 | 0.682 | 0.5236 | Yes |
| 137 | CTNNAL1 |  |  | 405 | 0.676 | 0.5237 | Yes |
| 138 | BRIX1 |  |  | 410 | 0.672 | 0.5254 | Yes |
| 139 | CCDC34 |  |  | 411 | 0.672 | 0.5282 | Yes |
| 140 | PA2G4 |  |  | 412 | 0.672 | 0.5309 | Yes |
| 141 | SAE1 |  |  | 418 | 0.669 | 0.5323 | Yes |
| 142 | KPNA2 |  |  | 420 | 0.669 | 0.5348 | Yes |
| 143 | RAN |  |  | 424 | 0.667 | 0.5368 | Yes |
| 144 | SMC3 |  |  | 425 | 0.667 | 0.5395 | Yes |
| 145 | PMAIP1 |  |  | 426 | 0.666 | 0.5422 | Yes |
| 146 | HNRNPAB |  |  | 435 | 0.659 | 0.5428 | Yes |
| 147 | ZWILCH |  |  | 436 | 0.659 | 0.5455 | Yes |
| 148 | NXT1 |  |  | 438 | 0.657 | 0.5479 | Yes |
| 149 | BUB3 |  |  | 441 | 0.657 | 0.5501 | Yes |
| 150 | PARP2 |  |  | 451 | 0.650 | 0.5504 | Yes |
| 151 | NUDT15 |  |  | 461 | 0.646 | 0.5507 | Yes |
| 152 | SNRPB |  |  | 467 | 0.642 | 0.5520 | Yes |
| 153 | DCTPP1 |  |  | 477 | 0.638 | 0.5522 | Yes |
| 154 | SSRP1 |  |  | 478 | 0.638 | 0.5548 | Yes |
| 155 | CKLF |  |  | 482 | 0.637 | 0.5567 | Yes |
| 156 | PGP |  |  | 492 | 0.631 | 0.5569 | Yes |
| 157 | RHEB |  |  | 500 | 0.629 | 0.5576 | Yes |
| 158 | POP7 |  |  | 502 | 0.628 | 0.5599 | Yes |
| 159 | BIRC5 |  |  | 505 | 0.628 | 0.5620 | Yes |
| 160 | SNRPD1 |  |  | 523 | 0.615 | 0.5600 | Yes |
| 161 | RPA3 |  |  | 532 | 0.611 | 0.5604 | Yes |
| 162 | BTG3 |  |  | 540 | 0.607 | 0.5611 | Yes |
| 163 | FHL2 |  |  | 541 | 0.607 | 0.5636 | Yes |
| 164 | HSPA8 |  |  | 547 | 0.603 | 0.5647 | Yes |
| 165 | MIS18BP1 |  |  | 574 | 0.588 | 0.5603 | Yes |
| 166 | CENPF |  |  | 575 | 0.588 | 0.5627 | Yes |
| 167 | RGCC |  |  | 600 | 0.578 | 0.5588 | Yes |
| 168 | EXOSC8 |  |  | 604 | 0.576 | 0.5603 | Yes |
| 169 | HSPD1 |  |  | 606 | 0.576 | 0.5624 | Yes |
| 170 | SMS |  |  | 608 | 0.575 | 0.5645 | Yes |
| 171 | SNRNP40 |  |  | 609 | 0.574 | 0.5669 | Yes |
| 172 | FBL |  |  | 612 | 0.573 | 0.5687 | Yes |
| 173 | CACYBP |  |  | 613 | 0.573 | 0.5710 | Yes |
| 174 | TUBG1 |  |  | 617 | 0.570 | 0.5726 | Yes |
| 175 | SMC1A |  |  | 618 | 0.570 | 0.5749 | Yes |
| 176 | CKAP2 |  |  | 619 | 0.569 | 0.5772 | Yes |
| 177 | TNFRSF12A |  |  | 629 | 0.566 | 0.5772 | Yes |
| 178 | HAT1 |  |  | 632 | 0.565 | 0.5790 | Yes |
| 179 | NCL |  |  | 638 | 0.560 | 0.5800 | Yes |
| 180 | HAUS1 |  |  | 649 | 0.552 | 0.5796 | Yes |
| 181 | NUSAP1 |  |  | 654 | 0.550 | 0.5808 | Yes |
| 182 | DCK |  |  | 661 | 0.548 | 0.5815 | Yes |
| 183 | PAICS |  |  | 668 | 0.546 | 0.5821 | Yes |
| 184 | H2AFX |  |  | 671 | 0.544 | 0.5838 | Yes |
| 185 | TUBB |  |  | 678 | 0.538 | 0.5845 | Yes |
| 186 | SLC25A5 |  |  | 680 | 0.537 | 0.5864 | Yes |
| 187 | PRKDC |  |  | 681 | 0.537 | 0.5886 | Yes |
| 188 | MRPL17 |  |  | 682 | 0.537 | 0.5908 | Yes |
| 189 | NETO2 |  |  | 740 | 0.519 | 0.5779 | No |
| 190 | NASP |  |  | 741 | 0.518 | 0.5801 | No |
| 191 | PTGES3 |  |  | 763 | 0.512 | 0.5767 | No |
| 192 | CMC2 |  |  | 785 | 0.503 | 0.5732 | No |
| 193 | EIF4EBP1 |  |  | 805 | 0.496 | 0.5702 | No |
| 194 | ACYP1 |  |  | 834 | 0.489 | 0.5649 | No |
| 195 | TUBA1B |  |  | 862 | 0.481 | 0.5598 | No |
| 196 | RRS1 |  |  | 866 | 0.480 | 0.5609 | No |
| 197 | ARL6IP6 |  |  | 880 | 0.476 | 0.5595 | No |
| 198 | DERA |  |  | 886 | 0.474 | 0.5601 | No |
| 199 | CISD2 |  |  | 891 | 0.473 | 0.5610 | No |
| 200 | CDKN2D |  |  | 911 | 0.468 | 0.5579 | No |
| 201 | HIRIP3 |  |  | 936 | 0.462 | 0.5535 | No |
| 202 | SRSF2 |  |  | 941 | 0.461 | 0.5544 | No |
| 203 | SNRPA1 |  |  | 954 | 0.458 | 0.5531 | No |
| 204 | KIF22 |  |  | 975 | 0.450 | 0.5497 | No |
| 205 | HSP90AA1 |  |  | 1008 | 0.443 | 0.5431 | No |
| 206 | PDCD5 |  |  | 1051 | 0.428 | 0.5338 | No |
| 207 | SRSF7 |  |  | 1071 | 0.424 | 0.5306 | No |
| 208 | HIST1H2AM |  |  | 1078 | 0.422 | 0.5307 | No |
| 209 | CDKN1A |  |  | 1088 | 0.419 | 0.5301 | No |
| 210 | CYCS |  |  | 1113 | 0.415 | 0.5255 | No |
| 211 | HNRNPF |  |  | 1128 | 0.412 | 0.5235 | No |
| 212 | POLR3K |  |  | 1140 | 0.410 | 0.5223 | No |
| 213 | HNRNPD |  |  | 1177 | 0.401 | 0.5144 | No |
| 214 | SNRPE |  |  | 1185 | 0.399 | 0.5142 | No |
| 215 | EIF1AX |  |  | 1198 | 0.397 | 0.5127 | No |
| 216 | MZT1 |  |  | 1219 | 0.393 | 0.5091 | No |
| 217 | PTMA |  |  | 1233 | 0.390 | 0.5073 | No |
| 218 | PSMC3 |  |  | 1241 | 0.389 | 0.5070 | No |
| 219 | STMN1 |  |  | 1249 | 0.387 | 0.5068 | No |
| 220 | HES4 |  |  | 1250 | 0.387 | 0.5083 | No |
| 221 | RMI2 |  |  | 1279 | 0.381 | 0.5026 | No |
| 222 | MAGOHB |  |  | 1336 | 0.369 | 0.4894 | No |
| 223 | RNASEH2A |  |  | 1341 | 0.369 | 0.4898 | No |
| 224 | PARP1 |  |  | 1431 | 0.351 | 0.4679 | No |
| 225 | PRELID1 |  |  | 1460 | 0.346 | 0.4620 | No |
| 226 | PFDN2 |  |  | 1479 | 0.343 | 0.4586 | No |
| 227 | IER2 |  |  | 1487 | 0.342 | 0.4582 | No |
| 228 | TMEM237 |  |  | 1576 | 0.328 | 0.4364 | No |
| 229 | CALM2 |  |  | 1625 | 0.320 | 0.4251 | No |
| 230 | PXMP2 |  |  | 1665 | 0.315 | 0.4162 | No |
| 231 | LSM4 |  |  | 1676 | 0.313 | 0.4149 | No |
| 232 | HMGB1 |  |  | 1720 | 0.308 | 0.4048 | No |
| 233 | CTGF |  |  | 1782 | 0.301 | 0.3900 | No |
| 234 | SRSF3 |  |  | 1834 | 0.294 | 0.3779 | No |
| 235 | RNPS1 |  |  | 1841 | 0.293 | 0.3775 | No |
| 236 | CMTM7 |  |  | 1845 | 0.293 | 0.3779 | No |
| 237 | RPL22L1 |  |  | 1966 | 0.277 | 0.3475 | No |
| 238 | MRPL18 |  |  | 1972 | 0.276 | 0.3473 | No |
| 239 | MXD3 |  |  | 2156 | 0.256 | 0.3003 | No |
| 240 | RAB13 |  |  | 2381 | -0.268 | 0.2426 | No |
| 241 | TMEM158 |  |  | 2476 | -0.280 | 0.2191 | No |
| 242 | MAGED2 |  |  | 2571 | -0.297 | 0.1956 | No |
| 243 | PFDN5 |  |  | 2602 | -0.301 | 0.1890 | No |
| 244 | MLLT11 |  |  | 2760 | -0.328 | 0.1491 | No |
| 245 | IGFBP5 |  |  | 2820 | -0.339 | 0.1350 | No |
| 246 | POLD4 |  |  | 2936 | -0.362 | 0.1063 | No |
| 247 | TMEM59 |  |  | 2960 | -0.366 | 0.1018 | No |
| 248 | ATP6V1G1 |  |  | 2968 | -0.367 | 0.1014 | No |
| 249 | MIAT |  |  | 2996 | -0.371 | 0.0959 | No |
| 250 | ID3 |  |  | 3112 | -0.402 | 0.0673 | No |
| 251 | RNF19A |  |  | 3137 | -0.411 | 0.0627 | No |
| 252 | COMMD6 |  |  | 3174 | -0.421 | 0.0550 | No |
| 253 | SOX9 |  |  | 3230 | -0.435 | 0.0423 | No |
| 254 | LAPTM4A |  |  | 3231 | -0.436 | 0.0441 | No |
| 255 | SDCBP |  |  | 3257 | -0.442 | 0.0393 | No |
| 256 | PLD3 |  |  | 3285 | -0.452 | 0.0341 | No |
| 257 | EIF4A2 |  |  | 3289 | -0.453 | 0.0352 | No |
| 258 | RABAC1 |  |  | 3388 | -0.493 | 0.0115 | No |
| 259 | ARL6IP5 |  |  | 3529 | -0.557 | -0.0230 | No |
| 260 | WDR83OS |  |  | 3533 | -0.562 | -0.0215 | No |
| 261 | IFITM3 |  |  | 3578 | -0.589 | -0.0306 | No |
| 262 | YPEL5 |  |  | 3603 | -0.605 | -0.0345 | No |
| 263 | PHLDA1 |  |  | 3622 | -0.617 | -0.0367 | No |
| 264 | SAT1 |  |  | 3659 | -0.653 | -0.0434 | No |
| 265 | NDFIP1 |  |  | 3687 | -0.672 | -0.0478 | No |
| 266 | SCD5 |  |  | 3765 | -0.751 | -0.0649 | No |
| 267 | PLAT |  |  | 3766 | -0.752 | -0.0618 | No |
| 268 | PRCP |  |  | 3775 | -0.766 | -0.0608 | No |
| 269 | ENC1 |  |  | 3787 | -0.774 | -0.0605 | No |
| 270 | BEX4 |  |  | 3796 | -0.785 | -0.0594 | No |
| 271 | SOX4 |  |  | 3797 | -0.786 | -0.0562 | No |
| 272 | TTYH1 |  |  | 3807 | -0.795 | -0.0553 | No |
| 273 | LYPD1 |  |  | 3844 | -0.834 | -0.0614 | No |
| 274 | PSAP |  |  | 3882 | -0.907 | -0.0674 | No |
| 275 | ZFAND6 |  |  | 3904 | -0.952 | -0.0690 | No |
| 276 | TM7SF2 |  |  | 3918 | -0.999 | -0.0683 | No |
| 277 | PCSK1N |  |  | 3987 | -1.179 | -0.0813 | No |
| 278 | ITM2B |  |  | 3995 | -1.225 | -0.0781 | No |
| 279 | CDH2 |  |  | 3998 | -1.233 | -0.0736 | No |
| 280 | GPM6A |  |  | 4002 | -1.248 | -0.0693 | No |
| 281 | SESN3 |  |  | 4003 | -1.260 | -0.0641 | No |
| 282 | TUBA1A |  |  | 4006 | -1.302 | -0.0593 | No |
| 283 | IGFBP3 |  |  | 4058 | -1.726 | -0.0657 | No |
| 284 | CLU |  |  | 4069 | -1.908 | -0.0605 | No |
| 285 | SCRG1 |  |  | 4070 | -2.021 | -0.0522 | No |
| 286 | BEX1 |  |  | 4072 | -2.034 | -0.0441 | No |
| 287 | S100B |  |  | 4081 | -2.383 | -0.0365 | No |
| 288 | GPM6B |  |  | 4083 | -2.435 | -0.0268 | No |
| 289 | FABP7 |  |  | 4095 | -3.351 | -0.0160 | No |
| 290 | PTPRZ1 |  |  | 4097 | -4.027 | 0.0003 | No |
Table: GSEA details [plain text format]

  

Fig 2: FELDMAN\_2020\_S-G2: Random ES distribution      
 Gene set null distribution of ES for **FELDMAN\_2020\_S-G2**
